# Patients’ perspectives on smartphone-based clinical photography in resource-constrained settings: a cross-sectional study

**DOI:** 10.1097/MS9.0000000000005626

**Published:** 2026-09-30

**Authors:** Urmila Gurung, Ushant Acharya, Khagendra Bhandari, Kiran Bhusal

**Affiliations:** Department of ENT-HNS, Maharajgunj Medical Campus-TU Teaching Hospital, Institute of Medicine, Maharajgunj, Kathmandu, Nepal

**Keywords:** patient preference, photography, questionnaire, smartphone, survey

## Abstract

**Background:**

The use of smartphones in clinical photography is widespread, particularly where a dedicated clinical photography tool is unavailable. This study aimed to gain insight into patients’ perspectives on the use of smartphones for clinical photography in resource-constrained settings.

**Methods:**

This cross-sectional study was conducted from July to September 2024 in the Department of Ear, Nose, and Throat – Head and Neck Surgery of a tertiary referral hospital in Nepal. A 12-question self-administered questionnaire was distributed to 196 patients aged 16 years or older who visited the outpatient department during the study period and agreed to participate. The questionnaire consisted of questions related to sociodemographic characteristics, previous experience with clinical photography, consent, preferred photographer, agreeable purposes for clinical photography, and reasons for refusal.

**Results:**

Of 196 participants, 144 [73.47%, 95% confidence interval (CI), 66.9–79.2%] were receptive to being photographed with a smartphone, preferably by the attending doctor [119/144 (82.64%)]. The need for consent for such a purpose was expressed by 145 participants (73.98%, 95% CI, 67.4–79.6%). Once photographed, 113 participants (78.47%) preferred to view the photograph. This preference was significantly higher among females [54/62 (87.10%)] than among males [59/82 (71.95%, 95% CI, 61.4–80.5%)] (*P* = 0.047) and among the educated group (*P* = 0.002). Patients were more open to non-identifiable photographs [109/144 (75.69%)] than to identifiable photographs [102/144 (70.83%)]. The acceptance of identifiable photographs increased if it was perceived to benefit their treatment rather than for commercial use.

**Conclusions:**

Most patients in this resource-constrained setting were accepting of smartphone-based clinical photography, provided that it was done with consent and by the attending doctor. Preferences leaned strongly toward non-identifiable images and transparency in use, underscoring the importance of ethical and respectful photographic practices in clinical settings.

## Introduction

Clinical photography is an indispensable tool that facilitates the documentation of a patient’s clinical condition during treatment, which further assists decision-making^[^1,2^]^. Its use also extends to non-therapeutic purposes such as medical education, research, and publications^[^1^]^, thereby increasing its value in research^[^3^]^.

HIGHLIGHTS

- This cross-sectional study aims to gain insight into patients’ perspectives on the use of smartphones for clinical photography in resource-constrained settings.
- Most patients in this resource-constrained setting were accepting of smartphone-based clinical photography, provided it was done with consent and by the attending physician.
- Preferences leaned strongly toward non-identifiable images and transparency in use, underscoring the importance of ethical and respectful photographic practices in clinical settings.

Hospitals in developed countries are equipped with hospital cameras and trained medical photographers for photo documentation and appropriate storage of these images^[^1^]^. However, with the integration of high-quality digital cameras into modern smartphones, their use in clinical photography has become inevitable^[^4^]^. They are considered convenient and time-efficient, allowing easier communication with other doctors and personal reference in the future^[^1^]^.

Based on survey responses from 103 surgeons and 44 residents of the Canadian Society of Plastic Surgeons, 89.1% acknowledged using their smartphones to take patients’ pictures^[^5^]^. A similar practice was noted among 93% of doctors across different medical specialties at a Portuguese hospital^[^3^]^. This practice has been rampant, more so since the COVID-19 pandemic, when virtual consultations with images of health conditions became the norm^[^2^]^.

Compared with a standard camera, taking clinical photos with a smartphone may appear less professional, and the images may be of lower quality, such as those affected by poor lighting^[^1^]^. Nevertheless, in a resource-constrained setting in an underserved country with no dedicated clinical photography setup or team, using a smartphone remains a viable option.

Despite the ease of using smartphones for clinical photography, concerns about consent, data storage, and confidentiality breaches might raise ethical or medico-legal issues^[^6^]^. More often than not, there is a tendency to store these photographs on doctors’ personal devices and to transfer them through mobile applications, which may risk unintentionally revealing these photographs to an unintended recipient^[^4^]^.

The patient’s comfort and perspective on this are seldom given due importance. Understanding the patient’s perspective is crucial for improving patient experience and maintaining a positive patient–doctor relationship^[^7^]^. This study thus aimed to comprehend patients’ acceptance levels and preferences, to identify concerns related to the use of smartphones for clinical photography in this context, and to explore their implications in clinical practice. This is all the more relevant given the lack of standalone, formal local hospital guidelines for clinical photography using smartphones.

## Methods

This cross-sectional study was conducted at the Outpatient Department (OPD) of the Department of Ear, Nose, and Throat–Head and Neck Surgery (ENT) at a tertiary referral hospital from July to September 2024. Ethical approval was obtained from the Institutional Review Committee [340(6–11)*E*^2^ 078/079] before commencing the study.

The calculated sample size was 196, using Cochran’s formula, with a 95% confidence level, a 50% population proportion, and a 7% margin of error, considering the cross-sectional study design assessing proportions and percentages. A 7% margin of error was chosen due to limited recruitment time and resources. Convenience sampling was used, in which consecutive patients aged 16 years and above who attended the OPD during the study period and consented to participate in the study were included, irrespective of clinical photography experience. Clinical photographs in the study referred to still, color images of a visible anatomical region (head, neck, ear, or nose) captured using smartphone cameras for various purposes such as obtaining a second opinion from another doctor, hospital records, illustrating other patients with similar illnesses, medical teaching, medical journals, public awareness websites, and commercial use.

Written consent was obtained by the corresponding author from all enrolled participants. Additional assent from minors and consent from their parents were obtained for participants under 18 years of age.

A self-administered 12-question written questionnaire adapted from Nair *et al*^[^7^]^ was used. It included questions related to sociodemographics, prior experience of being photographed for clinical photography purposes, consent, preferred photographer, agreeable purposes for clinical photography, and reasons for refusal. The questionnaire was translated from English into Nepali using forward-backward translation methodology: two independent bilingual researchers performed forward translation, and a third independent bilingual researcher performed back-translation. A pilot study was conducted among the first 30 patients to ensure that the questions were understandable.

Those who consented to participate in the study were asked to complete a self-administered, 12-question written questionnaire (Supplemental Digital Content Material, available at: https://links.lww.com/MS9/B393). For illiterate patients, the corresponding author read the questions aloud and recorded their responses. Multiple answers were allowed for questions with multiple options. Responses with missing data were excluded from the study.

### Data analysis

Statistical analysis was performed using SPSS version 24. The Cronbach’s alpha of the responses to the Nepalese-translated questionnaire was 0.7, indicating its reliability. Categorical variables were compared using the chi-square test, Fisher’s exact test, or the Monte Carlo exact test. The 95% confidence interval (CI) was calculated using the Wilson score method, and effect sizes for associations were reported as Cramér’s V, with interpretation based on Cohen’s criteria (for degrees of freedom = 1: small ≥ 0.10, medium ≥ 0.30, large ≥ 0.50)^[^8^]^. No correction for multiple comparisons (e.g., Bonferroni) was applied, as the analyses were largely exploratory and hypothesis-generating rather than confirmatory. A *P*-value less than 0.05 was considered statistically significant. The work has been reported in line with the STROCSS 2025 criteria^[^9^]^.

## Results

A total of 196 patients were enrolled in the study.

### Demography

Among the 196 patients, ages ranged from 16 to 84 years (mean 34.9 ± 14.65 years), of whom 117 (59.69%) were male and 79 (40.31%) were female. The majority were Hindu, married, and literate (Table 1).

**Table 1:** Demography of participants, consent, and reluctance to clinical photography (*N* = 196).

Demography	*n* (%)	Consent required	Consent not required	*P*-value	Cramér’s V	Receptive to clinical photography	Reluctant to clinical photography	*P*-value	Cramér’s V
Gender
Male	117 (59.7%)	85	32	0.726^a^	0.025	82	35	0.254^a^	0.082
Female	79 (40.3%)	60	19			62	17		
Age
≤45 years	161 (82.1%)	127	34	0.002^a^	0.225	118	43	1.000^a^	0.000
>45 years	35 (17.9%)	18	17			26	9		
Religion
Hindu	159 (81.1%)	120	39	0.203^b^	0.150	120	39	0.260^b^	0.144
Buddhist	25 (12.8%)	19	6			16	9		
Christian	9 (4.6%)	4	5			7	2		
Muslim	3 (1.5%)	2	1			1	2		
Marital Status
Married	113 (57.7%)	78	35	0.118^b^	0.150	84	29	0.911^b^	0.057
Unmarried	81 (41.3%)	66	15			59	22		
Widowed/divorced	2 (1.0%)	1	1			1	1		
Education Level
Illiterate	16 (8.2%)	5	11	<0.001^b^	0.363	9	7	0.182^b^	0.132
Secondary level	114 (58.2%)	80	34			83	31		
Undergraduate and above	66 (33.6%)	60	6			52	14		

^a^ Chi-square test.

^b^ Monte Carlo exact test.

### Perception on consent for clinical photography

Of the total study patients, 145 (73.98%) expressed the need for consent for smartphone-based clinical photography, with younger patients (age < 45 years) (chi-square test *P* = 0.002, Cramér’s V = 0.225, small effect) and those with higher education (undergraduate and above) (Fisher’s exact test *P* < 0.001, Cramér’s V = 0.363, medium effect) favoring it. However, there was no statistically significant difference based on gender, religion, or marital status (Table 1).

### Acceptance/receptive to clinical photography using a smartphone

Forty out of 196 participants (20.41%) had prior experience of being photographed with a smartphone for medical reasons in hospital or clinic settings. Among all participants, 144 (73.47%; 95% CI, 66.9–79.2%; margin of error, ±6.2% using the Wilson score method) were receptive to being photographed with a smartphone (Table 1). The preferred photographer in this context was most often the attending doctor (119/144, 82.64%), followed by nursing staff (24/144, 16.67%), and least often by medical students or paramedics (1/144, 0.69%).

Once photographed, 113 participants (78.47%; 95% CI, 71.1–84.4%) preferred to view the photograph. Among them, females [54/62; 87.10%; 95% CI, 76.6–93.3%] exceeded the number of males [59/82; 71.95%], with the difference being statistically significant (chi-square test, *P* = 0.047; Cramér’s V = 0.165, small effect). Education level also significantly predicted viewing preference (Monte Carlo *P* = 0.002; Cramér’s V = 0.299, small effect). Illiterate participants were substantially less likely to want to view their photographs [3/9 (33.3%; 95% CI, 12.1–64.6%)] compared to those with secondary [65/83 (78.3%; 95% CI, 68.3–85.8%)] or undergraduate [45/52 (86.5%; 95% CI, 74.7–93.3%)] education.

Multivariate logistic regression was conducted to assess independent predictors of acceptance. After controlling for age, gender, education, and previous photography experience, no variable independently predicted acceptance of clinical photography (Table 2).

**Table 2:** Multivariable analysis.

Variable	Category	*n* (%)	Crude odds ratio (95% CI)	Adjusted odds ratio (95% CI)	*P*-value*
Age	Continuous	196 (100.0)	1.02 (0.99–1.05)	1.00 (0.98–1.03)	0.769
Gender	Male	117 (59.7)	1.00 [Reference]	1.00 [Reference]	–
	Female	79 (40.3)	1.56 (0.80–3.03)	1.73 (0.87–3.43)	0.119
Education	Illiterate	16 (8.2)	1.00 [Reference]	1.00 [Reference]	–
	Secondary	114 (58.2)	2.08 (0.71–6.07)	2.08 (0.64–6.74)	0.223
	Undergraduate or above	66 (33.7)	2.89 (0.91–9.13)	2.99 (0.77–11.58)	0.113
Previous photography experience	No	156 (79.6)	1.00 [Reference]	1.00 [Reference]	–
	Yes	40 (20.4)	2.37 (0.93–6.03)	2.40 (0.92–6.26)	0.073

* Wald tests.

### Identifiable versus non-identifiable photographs

Depending on the purpose of clinical photography, 109 out of 144 (75.69%) preferred non-identifiable photographs, while 102 out of 144 (70.83%) were open to identifiable photographs. For non-identifiable photographs, 50% or more of participants were receptive to photographs for medical teaching, medical journals, and public awareness websites. Similarly, the majority were open to identifiable photographs intended to obtain a second opinion from another doctor, hospital records, and illustrations for other patients with similar illnesses (Table 3).

**Table 3:** Receptivity to clinical photography by intended purpose amongst patients open to clinical photography (*N* = 144).

Purpose	Identifiable photographs (%)	Non-identifiable photographs (%)
Documentation for hospital record	66 (45.8%) [37.9–54.0%]	52 (36.1%) [28.7–44.2%]
Seeking second opinion from another doctor	77 (53.5%) [45.3–61.4%]	59 (41.0%) [33.3–49.1%]
Teaching medical students and health professionals	44 (30.6%) [23.6–38.5%]	85 (59.0%) [50.9–66.7%]
Medical journals/presentation/research	42 (29.2%) [22.4–37.1%]	72 (50.0%) [41.9–58.1%]
Medical websites for public awareness	35 (24.3%) [18.0–31.9%]	76 (52.8%) [44.7–60.8%]
Illustrations to other patients with similar illness	68 (47.2%) [39.2–55.3%]	69 (47.9%) [39.9–56.0%]
Commercial use/advertisement	25 (17.4%) [12.0–24.4%]	71 (49.3%) [41.3–57.4%]

### Reluctance to clinical photography

Those reluctant to undergo clinical photography amounted to 26.53% (52 out of 196; 95% CI, 20.8–33.1%), of whom 35 (67.31%) were male and 17 (32.69%) were female, most of whom (*n* = 43; 82.69%) were younger than 45 years. Most were literate, with 31 (59.62%) having completed secondary-level education and 14 (26.92%) being undergraduates. The majority, 29 (55.77%), were married (Table 1).

The key concerns leading to hesitancy about clinical photographs were the potential loss of privacy or confidentiality (42/52, 80.77%) and the circulation of photographs on social media (32/52, 61.54%) (Table 4). A stratified analysis of reasons for reluctance by gender, age group, and education level was conducted (Table 4). No significant differences were found by gender or age. However, education level was significantly associated with concerns about adverse effects on future careers (*P* = 0.017).

**Table 4:** Reason for reluctance to clinical photography (*n* = 52).

Reason for reluctance to clinical photography	Number (%)	*P*-value*
Circulation of photograph on social media	32 (61.5%)	
Gender
Male	21	1.000
Female	11	
Age (in years)
≤45	27	0.719
>45	5	
Education
Illiterate	4	0.950
Secondary education	19	
Undergraduate and above	9	
Psychological/Emotional effect	21 (40.4%)	
Gender
Male	15	0.765
Female	6	
Age (in years)
≤45	18	0.724
>45	3	
Education
Illiterate	2	0.790
Secondary education	13	
Undergraduate and above	6	
Loss of privacy/confidentiality	42 (80.8%)	
Gender
Male	28	1.000
Female	14	
Age (in years)
≤45	35	1.000
>45	7	
Education
Illiterate	5	0.736
Secondary education	25	
Undergraduate and above	12	
Bad impression/ experience from previous inappropriate use of one’s or another patient’s clinical photograph	18 (34.6%)	
Gender
Male	13	0.758
Female	5	
Age (in years)
≤45	14	0.702
>45	4	
Education
Illiterate	4	0.265
Secondary education	11	
Undergraduate and above	3	
Adverse effect on future/career	20 (38.5%)	
Gender
Male	15	0.383
Female	5	
Age (in years)
≤45	15	0.280
>45	5	
Education
Illiterate	4	0.017
Secondary education	15	
Undergraduate and above	1	

* Chi-square test/Fisher’s exact test.

## Discussion

The use of smartphones for clinical photography is widespread, especially where a dedicated clinical photography tool is unavailable. This study aimed to gain insight into patients’ perspectives on the use of smartphones for clinical photography in resource-constrained settings.

### Acceptance/receptive to clinical photography using a smartphone

A majority of patients (73.47%) in this study were receptive to clinical photography using a smartphone. This high level of acceptance resonated with the findings (68%) of a survey conducted by Nair *et al*^[^7^]^ in an oculoplastic clinic. Most participants considered it useful for documenting their medical conditions, for medical education (89.3%), and for a better understanding of their medical condition and treatment (67.9%). In their study, approval was higher among those aged less than 40 years and among smartphone owners. This is probably due to the general openness of patients to the use of smartphones in healthcare services, including clinical photography, in today’s digital age. Wyatt *et al*^[^10^]^ found that patients were unconcerned whether a mobile device or a professional camera was used for clinical photography. In fact, 74% of them were willing to recommend clinical photography to others, and 67% felt that clinical photos supported their clinical care.

### Consent

In our study, a large proportion of patients (*n* = 145, 73.98%) expressed the need for consent to use smartphones for taking clinical photographs. This was particularly significant among younger and more educated patients (*P* < 0.001). The findings mirrored studies conducted in culturally different settings. Nair *et al*^[^7^]^ also observed that 72% favored obtaining consent before the photographs were used, with 84% agreeing that consent was needed the first time but not for subsequent uses. In a survey of patients in Minnesota, the United States, who had been previously photographed across various specialties, 79% expressed the need for consent, with only 27% favoring written consent, while 21% did not feel the need for any specific consent process^[^10^]^. This showed that most patients across different studies were aware of the need for prior consent of some form, indicating their understanding of autonomy. Nonetheless, some patients appeared unconcerned. This could be due to their being uninformed of the possibility of photos being publicly accessible, especially when published in open-access medical journals^[^11^]^.

Cura *et al*^[^3^]^ perceived a difference in perceptions toward consent among doctors based on purpose. Formal consent, either written alone or with oral consent, was obtained when using photographs for research, publication, or medical conferences, while leniency was shown toward only verbal consent for clinical reasons, such as documenting clinical progress or seeking a second opinion. It is not uncommon to find clinicians resorting to an informal approach, typically verbal consent, possibly due to a lack of awareness of the consequences of digitalization associated with the academic publication of patients’ photographs^[^11^]^.

Respecting the patient’s autonomy is important^[^12^]^. Obtaining written consent before taking photographs is considered good clinical practice. The consent should specify the purpose and potential uses of these photographs in medical journals, books, presentations, websites, and patient information leaflets^[^2,13^]^. Cultural differences in consent – such as situations where female patients need their husband’s permission to be photographed^[^12^]^ – as well as issues related to literacy hurdles and inability to comprehend the information provided^[^14^]^, should also be taken into account. Additionally, consent should address the potential risk of these images being publicly available and the fact that they cannot be completely retracted once they are in the public domain^[^13,15^]^. Interestingly, the vast majority of patients continue to consent irrespective of this risk^[^13^]^. Amirian *et al*^[^12^]^ noted that patients with lower educational levels were more likely to participate, attributing this to a possible limited understanding of patients’ rights.

### Choice of preferred photographer

In our study, there were differences in the choices of preferred photographers, with most participants preferring the treating doctor. A similar finding was reported in a study by Nair *et al*^[^7^]^ in which treating doctors as photographers were preferred over health personnel such as medical students, interns, nurses, or even medical photographers. Likewise, a majority approved of doctors and trainee doctors having access to their photographs, unlike other healthcare personnel. Amirian *et al*^[^12^]^ also noted that patients trusted those doctors directly involved in their treatment to access their digital photos. This was likely to avoid unnecessary exposure of sensitive images to unauthorized personnel. Most (*n* = 113, 78.47%) expressed a desire to view the photographs, with females outnumbering males. This indicated that patients needed assurance to understand how they were portrayed in clinical photography. Showing photos intended for publication to patients when seeking their consent is best practice^[^11,16^]^.

### Preferences for identifiable vs. Non-identifiable photographs

In our study, 102 patients (70.83%) approved identifiable photographs only under certain circumstances, primarily seeking a second opinion (*n* = 77, 53.47%) or clinical documentation (*n* = 66, 45.83%), with less willingness noted for commercial use or advertising (*n* = 25, 17.36%). However, regarding non-identifiable photographs, patients were more open to purposes that cater to a broader audience, such as education, public awareness, public health websites, medical teaching, and medical journals. This suggests that patients are willing to permit identifiable photographs when doing so benefits their treatment, unlike with commercial use, where they may feel exposed or exploited, and similar findings have been noted in other studies as well^[^6,10^]^. The apprehension is understandable, as posting in the public domain is irreversible, and the reuse of such photographs can occur without permission, so it is crucial to anonymize identities when using photos for these purposes. However, this can be tricky due to a lack of relevant standard guidelines^[^16^]^, and identifiability is at times unavoidable in certain specialties such as dermatology, dentistry, and cosmetic surgery^[^11,17^]^, and in such contexts anonymization may not be necessary if proper consent is obtained^[^11^]^.

### Implications for healthcare practice

In this digital age, the use of smartphones in the healthcare system has become inevitable and widely accepted by patients. The use of a hospital-owned phone, secure mobile applications in different hospital settings, or work mobile phones could be an alternative to address privacy concerns^[^4,18^]^. In cases where personal devices are used, they should have strict electronic security measures, and the photo should be deleted at the earliest^[^18^]^.

Interestingly, awareness of the importance of obtaining written informed consent for the publication of clinical photography is scarce even among health professionals. In addition, even journal guidelines are inconsistent^[^19^]^ more so for online medical journals^[^11^]^. There is a need for clear ethical guidelines that protect patients’ dignity, formulate a culturally appropriate consent process^[^14^]^, and govern the handling, storage, and dissemination of clinical images to protect patient confidentiality^[^4^]^. The onus falls on the healthcare professional responsible for taking the photograph to ensure these^[^3,11^]^. Hence, training and awareness among healthcare professionals, especially at induction in a new hospital network or setup^[^18^]^, are needed to maintain professionalism and protect patients’ rights to privacy^[^19^]^. This will help build trust in using technologies in healthcare.

There are several limitations in the study. This was a single-center study performed in an ENT department using convenience sampling, which limits the generalizability of our results to other clinical settings. Due to local cultural sensitivities, a binary gender framework was adopted, which may restrict the applicability of our findings in countries with greater acceptance of gender diversity. Our study is susceptible to selection and non-response biases, as the number of eligible patients who declined to participate was not recorded, and we cannot account for patients who could have chosen to opt out due to their dislike of clinical photography. This could consequently have increased the acceptance rate. The data-collection constraints introduced potential bias; a literacy barrier required the researcher to assist some illiterate patients in completing the questionnaire, leading to interviewer bias. The study lacks an in-depth discussion of the local cultural context, which may be relevant to patient’s attitudes toward privacy and consent. However, it provides a perspective on patients who, if photographed, often require part or all of the face to be photographed, a situation that is likely to prompt hesitancy. This could provide a basis for future research involving patients of different specialties, as well as the formulation and implementation of relevant protocols.

## Conclusions

Most patients in this resource-constrained setting were accepting of smartphone-based clinical photography, provided it was done with consent and by the attending doctor. Preferences leaned strongly toward non-identifiable images and transparency in usage, underscoring the importance of ethical and respectful photographic practices in clinical settings. To ensure ethical practice, it is recommended to

1. develop culturally appropriate informed consent,
2. ensure the treating doctor is the photographer,
3. anonymize clinical photographs as far as possible,
4. allow patients to view the image after being photographed,
5. ensure secure storage and transmission of clinical photographs to avoid data security breach,
6. develop and implement institutional and national guidelines and provide training for healthcare professionals on ethical photography practices.

## Data Availability

The data are available on reasonable request.

## Research registration unique identifying number (UIN)

This was a non-interventional, cross-sectional observational survey and did not involve any intervention or clinical trial. The study was therefore not registered as a clinical trial, and no clinical trial registration number was obtained.

## Assistance with the study

Shishir Pokhrel: obtaining ethical approval.
